# Modeling of Poly(Ethylene Terephthalate) Homogeneous Glycolysis Kinetics

**DOI:** 10.3390/polym15143146

**Published:** 2023-07-24

**Authors:** Kirill A. Kirshanov, Roman V. Toms, Mikhail S. Balashov, Sergey S. Golubkov, Pavel V. Melnikov, Alexander Yu. Gervald

**Affiliations:** 1 M.V. Lomonosov Institute of Fine Chemical Technologies, MIREA—Russian Technological University, Moscow 119571, Russia; kirill_kirshanov@mail.ru (K.A.K.); toms.roman@gmail.com (R.V.T.); diplomglue@gmail.com (M.S.B.); gervald@bk.ru (A.Y.G.); 2 A.N. Nesmeyanov Institute of Organoelement Compounds, Russian Academy of Sciences, 28 Vavilova Str., Moscow 119334, Russia; golserg97@yandex.ru

**Keywords:** PET, polyethylene terephthalate, oligoethylene terephthalates, bis(2-hydroxyethyl) terephthalate, ethylene glycol, chemical recycling, transesterification, homogeneous glycolysis

## Abstract

Polymer composites with various recycled poly(ethylene terephthalate)-based (PET-based) polyester matrices (poly(ethylene terephthalate), copolyesters, and unsaturated polyester resins), similar in properties to the primary ones, can be obtained based on PET glycolysis products after purification. PET glycolysis allows one to obtain bis(2-hydroxyethyl) terephthalate and oligo(ethylene terephthalates) with various molecular weights. A kinetic model of poly(ethylene terephthalate) homogeneous glycolysis under the combined or separate action of oligo(ethylene terephthalates), bis(2-hydroxyethyl) terephthalate, and ethylene glycol is proposed. The model takes into account the interaction of bound, terminal, and free ethylene glycol molecules in the PET feedstock and the glycolysis agent. Experimental data were obtained on the molecular weight distribution of poly(ethylene terephthalate) glycolysis products and the content of bis(2-hydroxyethyl) terephthalate monomer in them to verify the model. Homogeneous glycolysis of PET was carried out at atmospheric pressure in dimethyl sulfoxide (DMSO) and N-methyl-2-pyrrolidone (NMP) solvents with catalyst based on antimony trioxide (Sb_2_O_3_) under the action of different agents: ethylene glycol at temperatures of 165 and 180 °C; bis(2-hydroxyethyl) terephthalate at 250 °C; and oligoethylene terephthalate with polycondensation degree 3 at 250 °C. Homogeneous step-by-step glycolysis under the successive action of the oligo(ethylene terephthalate) trimer, bis(2-hydroxyethyl) terephthalate, and ethylene glycol at temperatures of 250, 220, and 190 °C, respectively, was also studied. The composition of products was confirmed using FTIR spectroscopy. Molecular weight characteristics were determined using gel permeation chromatography (GPC), the content of bis(2-hydroxyethyl) terephthalate was determined via extraction with water at 60 °C. The developed kinetic model was found to be in agreement with the experimental data and it could be used further to predict the optimal conditions for homogeneous PET glycolysis and to obtain polymer-based composite materials with desired properties.

## 1. Introduction

Polyethylene terephthalate (PET) is one of the most widely used polymers in the world. The share of PET waste is about 25% of the total amount of plastic waste and is about 12% of the total amount of solid waste [1,2]. Sources of postconsumer polyethylene terephthalate are in various commodity forms, including, first of all, PET bottles [2], polyester textile fibers, used medical dressings, and polyester tire cord waste [3,4]. There are various approaches to the postconsumer utilization of polyethylene terephthalate, with chemical and mechanical (or physical) recycling being the main methods. Mechanical recycling is the re-extrusion of crystallized and dried PET particles. This method is most common in industry; however, polymer performance properties deteriorate due to the degradation in each processing cycle [1].

There are various routes to use PET waste disposal [5]. Polyethylene terephthalate, including recycled PET (rPET), is used not only as a packaging material and textile but also in the manufacturing of membranes for various purposes [1] and composites [6,7]. Polyethylene terephthalate is a common matrix for obtaining nanocomposites due to its outstanding properties: high thermal stability and strength, controlled crystallization rate, and recyclability. The most common nanomaterials used in PET-based composites are metal and metal oxide nanoparticles (ZnO, TiO_2_), nanotubes (single-wall and multiwall carbon nanotubes), and layered silicates (mostly montmorillonite) [7,8].

There are two key problems regarding the use of recycled polyethylene terephthalate as a matrix for nanocomposites. Firstly, although the addition of nanomaterials can significantly improve the properties of rPET [7], the properties of recycled poly(ethylene terephthalate)-based and rPET unsaturated polyester resin-based composites are inferior to those of composites based on virgin PET (vPET) [9,10,11]. rPET and rPET-based composites are known to have a lower Young’s modulus and tensile strength than vPET ones [12]. Secondly, a common method for obtaining nanocomposites based on polyethylene terephthalate is in situ polymerization, with the only possible way to use rPET as a matrix being melt mixing [8]. Moreover, in situ polymerization with the participation of oligoesters with hydroxyl groups makes it possible to obtain matrices of various compositions, including polylactide-based ones [13]. In addition, it should be noted that the use of rPET is undesirable in medical devices and products in contact with food.

A promising way to obtain monomers for the synthesis of PET, which could be similar in properties to the vPET, is chemical recycling [1,14]. Currently, a large number of studies are devoted to chemical recycling, and the main attention is paid to the investigation of the reaction mechanisms [15] and kinetics [16]. The most common methods are hydrolysis, methanolysis, glycolysis, and their combinations [16]. Glycolysis is of particular interest, since it allows one to obtain bis(2-hydroxyethyl) terephthalate, the latter being a monomer for the synthesis of PET, which is easily purifiable via recrystallization in water. The recycling by means of glycolysis is carried out under the action of compounds with at least two hydroxyl groups, and ethylene glycol is the most commonly used [3].

Both heterogeneous and homogeneous catalysts are used [17], with pseudohomogeneous catalysts being considered separately [18]. Nanoparticles and nanotubes [1], spinels [17], nanosheets [19,20], organocatalysts [21], and biomass-waste-derived catalysts [22] are used as heterogeneous catalysts. Zinc acetate is the most widespread among homogeneous catalysts [23,24,25]. The use of ethylene glycol with antimony trioxide is of particular interest, since it is a common polycondensation catalyst in the production of polyethylene terephthalate [26,27]. The use of ionic liquids in PET glycolysis is also widely investigated [28,29,30,31,32]. Furthermore, the reaction could be additionally accelerated by the use of microwave radiation [33,34], gamma radiation [35], and supercritical conditions combined with the addition of a catalyst.

Glycolysis is classified depending on the phase composition of the reaction mixture as heterogeneous [36,37,38] and homogeneous [3,6,23,24,39,40]. In heterogeneous PET glycolysis, the system contains two phases at the beginning of the process, namely liquid glycol and particles of polyethylene terephthalate distributed in it. Homogeneous glycolysis can be carried out in solution [23,24] or in a melt at a temperature above the pour point of PET [3,6,39,40].

The advantage of homogeneous glycolysis over heterogeneous is its high reaction rate. This makes homogeneous glycolysis a more energy efficient process. In addition, the high rate of reaching the equilibrium of the main reaction reduces the amount of impurities formed due to the irreversible side reactions. An important task for further study of homogeneous glycolysis both in solution and in melt is the development of its kinetic model, which takes into account the conditions of the process. The currently existing models take into account only the reactions of ester groups of PET and the resulting products of glycolysis with ethylene glycol, as well as reverse reactions [36,41]. This approach does not take into account the interaction of PET ester groups with terminal hydroxyl groups formed during the reaction. The development of a more general model will allow one to describe homogeneous glycolysis under the action of ethylene glycol more accurately and to simulate glycolysis under the action of bis(2-hydroxyethyl) terephthalate and oligoethylene terephthalates with terminal hydroxyl groups. Ultimately, it will become possible to carry out a directed synthesis of the material with desired properties, which is important, in particular, when creating polymer-based composites.

Models describing a reversible polycondensation reaction in the production of vPET [42,43,44,45] take into account the interactions between bound, terminal, and free ethylene glycol molecules in the reaction mixture. The aim of this work is to develop and verify a kinetic model of homogeneous poly(ethylene terephthalate) glycolysis that also takes into account these interactions.

## 2. Materials and Methods

### 2.1. Materials

Poly(ethylene terephthalate) was used in the form of clear PET flakes, which were obtained from postconsumer PET bottles (Tver Polymers Recycling Plant, Tver, Russia). Ethylene glycol, zinc acetate and antimony trioxide, dimethyl sulfoxide (DMSO), and N-methyl-2-pyrrolidone (NMP) were purchased from Sigma Aldrich (St. Louis, MO, USA) and were used without further purification.

### 2.2. Synthesis of Glycolytic Agents

Glycolytic agents were obtained in accordance with the procedures given in the article [3].

Bis(2-hydroxyethyl) terephthalate (BHET-1 sample) was obtained via heterogeneous glycolysis of PET flakes under the action of ethylene glycol with zinc acetate as a homogeneous catalyst in a mass ratio of 100:250:3, respectively. The process was carried out at a temperature of 190 °C and stirring at 200 rpm. The product was purified via recrystallization in water at 60 °C and dried.

Oligoethylene terephthalate with terminal hydroxyl groups and with a polycondensation degree equal to 3 (OET-1 sample) was obtained via heterogeneous glycolysis of PET flakes under the action of the obtained BHET-1 agent in a mass ratio of 100:250:3, respectively. The process was carried out under the same conditions as the preparation of BHET-1. The water-soluble components, namely ethylene glycol and zinc acetate, were extracted using water at 20 °C to avoid the simultaneous extraction of bis(2-hydroxyethyl) terephthalate.

### 2.3. Homogeneous Glycolysis

#### 2.3.1. Homogeneous Glycolysis under the Action of Ethylene Glycol in Solution

Homogeneous glycolysis in DMSO was carried out in accordance with the procedure described by Bo Liu et al. [23]. A solution of PET in DMSO in a mass ratio of 100:400 was mixed with a solution of antimony trioxide in ethylene glycol in a mass ratio of 7:200. The process was carried out at 180 °C and 200 rpm. Samples were taken every minute. The product was isolated via the extraction of DMSO, residual ethylene glycol, and catalyst using water at 20 °C.

Homogeneous glycolysis in NMP was carried out in accordance with the procedure described by Moncada et al. [24]. Similar to glycolysis in DMSO, a solution of PET in NMP in a mass ratio of 100:930 was mixed with a solution of antimony trioxide in ethylene glycol in a mass ratio of 13:270. Samples were taken every two minutes. The process was carried out at 165 °C and 200 rpm. The product was isolated via the extraction of NMP, residual ethylene glycol, and catalyst using water at 20 °C.

#### 2.3.2. Homogeneous Melt Glycolysis under the Action of BHET

Homogeneous glycolysis under the action of BHET was carried out in a reactor with reflux at a temperature of 250 °C and stirring at 100 rpm for 1.5 h. Antimony trioxide was mixed with ethylene glycol in a mass ratio of 1:1 to obtain a homogeneous transesterification catalyst. PET was preliminarily mixed with BHET-1 at 270 °C. PET, BHET-1 and antimony trioxide solution were used in mass ratios 100:130:2.3 (molar ratio PET:BHET = 1:1) and 100:390:4.9 (molar ratio PET:BHET = 1:3), respectively. The product was isolated similarly to the preparation of OET-1 (see Section 2.2).

#### 2.3.3. Homogeneous Melt Glycolysis under the Action of OET

To carry out homogeneous glycolysis, the mixing of PET and OET-1 at 270 °C was followed by the reaction in mass ratios of PET, OET, and Sb_2_O_3_ solution in ethylene glycol 100:110:2.1 (molar ratio PET:OET = 1:1) and 100:330:4.3 (molar ratio PET:OET = 1:3), respectively, at 250 °C and at 50 rpm for 1.5 h. The product was isolated similarly to the preparation of OET-1 (see Section 2.2).

#### 2.3.4. Step-By-Step Homogeneous Melt Glycolysis under the Action of OET, BHET, and Ethylene Glycol

**Figure 1:**
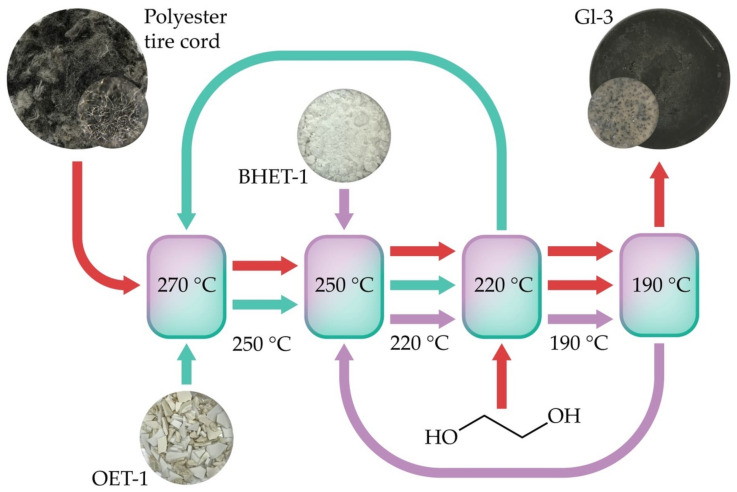
Homogeneous step-by-step glycolysis scheme [3].

Step-by-step glycolysis was carried out according to the procedure described previously [3]. The schematic diagram of the process is shown in Figure 1; the reaction conditions for each stage are given in Table 1 [3].

### 2.4. Characterization of PET, Glycolytic Agents, and Glycolysis Products

#### 2.4.1. Fourier Transform Infrared Spectroscopy (FTIR)

The chemical compositions of PET and the resulting oligoesters were confirmed using the position of the characteristic bands in the FTIR spectra. The spectra were obtained by means of the Spectrum 65 FT-IR spectrometer (Perkin Elmer, Waltham, MA, USA).

#### 2.4.2. Gel Permeation Chromatography (GPC)

Gel permeation chromatography (Gilson Inc., Middleton, WI, USA) with Agilent MIXED-E column (Agilent, Santa Clara, CA, USA), tetrahydrofuran as the mobile phase, and refractive index detector was used to determine the molecular weights of the component present in the analyzed samples. Measurements were made at the temperature of 25 °C and the flow rate of 1.0 mL/min. Narrowly dispersed polystyrene standards with Mp (peak molecular weight) 580; 1280; 2940; 10,110; and 28,770 g/mol and a polydispersity index of no more than 1.12 were used for calibration (Agilent, Santa Clara, CA, USA).

#### 2.4.3. Determination of Bis(2-Hydroxyethyl) Terephthalate Content

To determine the content of bis(2-hydroxyethyl) terephthalate monomer, the product, previously purified from water-soluble components at a temperature of 20 °C, was weighed and the masses of PET and/or OET-1, BHET-1 ($m_{PET}^{0},m_{OET-1}^{0},m_{BHET-1}^{0})$ were found according to the initial proportion. After that, the sample was immersed in water at a temperature of 60 °C for 1 h, and the resulting dispersion was filtered. A solution of BHET in water was cooled to 10 °C and filtered; the resulting BHET was dried and weighed ($m_{BHET})$. After that, the BHET yield was found using Equation (1) [23]:

(1) YBHET,experimental=mBHET254mPET0192+mOET−10212.7+mBHET−10254·100%

## 3. Results and Discussion

### 3.1. PET Flakes and Glycolytic Agents Characterization

The composition of PET flakes was confirmed by the presence of absorption bands in the range of PET characteristic frequencies: 1715 cm^–1^ (carbonyl, stretching), 1243 cm^–1^ (ester group, stretching), and 1176 and 1116 cm^–1^ (1,4-substituted ring) [6]. The correlation coefficient of the FTIR spectrum with the known spectrum of partially crystalline polyethylene terephthalate was 99%.

The obtained degree of polycondensation of glycolytic agents was determined by the ratio of bands corresponding to terminal hydroxyl groups (3350 cm^–1^) and carbonyl groups in the chain (1720 cm^–1^) of the FTIR spectrum and it was additionally confirmed by the number average molecular weights of the samples measured using GPC. The degrees of polycondensation of samples OET-1 and BHET-1 were 3.1 and 1.1, respectively, that corresponds to the data obtained previously [3].

### 3.2. Kinetic Model Development

A large number of reactions occur during high-temperature polycondensation of PET or glycolysis under the action of ethylene glycol, bis(2-hydroxyethyl) terephthalate, and oligoethylene terephthalates with terminal hydroxyl groups. The main ones include esterification and transesterification reactions, the formation of diethylene glycol or acetaldehyde side reactions, and thermal or thermo-oxidative degradation. A functional group model was used to simulate the process kinetics. The rate constants in the model are defined for the reactions between the functional end groups involved in each reaction, namely bonded, terminal ethylene glycol groups, and free ethylene glycol molecules. The abbreviations used in the kinetic model are given in Table 2.

A number of assumptions were made to simplify the model:

1. Polyesters within the same source (feedstock or agent) are in equilibrium. Therefore, the reactions occurring in them do not lead to a change in the molecular weight distribution. Under this assumption, the polydispersity index of polyester from a single source corresponds to the polyester obtained via ideal step-growth polymerization (Flory-Schulz distribution). Thus, the following reactions are not taken into account in the model:

EGhb+EGht↔EGhb+EGht

EGh+EGht↔EGh+EGht

EGlb+EGlt↔EGlb+EGlt

EGl+EGht↔EGl+EGht

**Figure 2:**
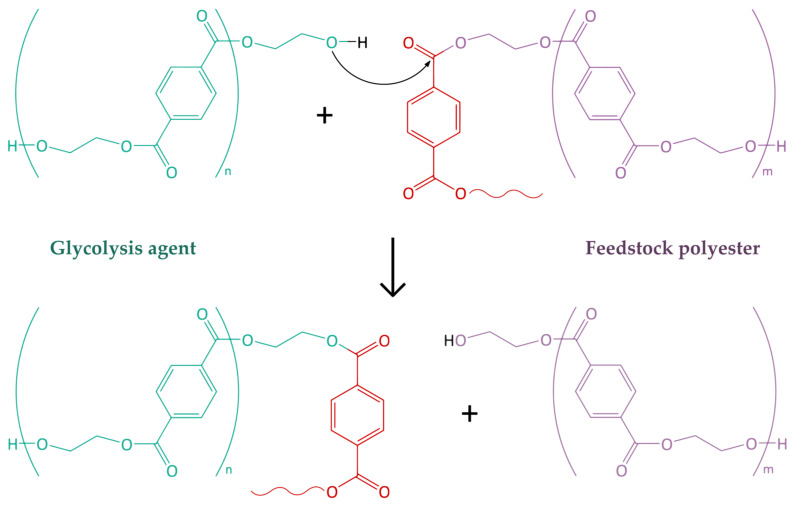
General scheme of polycondensation and glycolysis reactions under the action of ethylene glycol, BHET, and OET with terminal hydroxyl groups.

2. The concentration of the catalyst $\left[c_{cat}\right]$ in the reaction mixture is assumed to be constant. If this assumption is violated, the actual conversion of the process will be lower than the one calculated by the model. The effect of the catalyst is included in the effective constants [42,43] in Equations (2) and (3) (see below). The model was verified using the values for the antimony trioxide catalyst used;
3. All reactions under the action of terminal hydroxyethyl groups (EGt) proceed at the same rate, regardless of the chain lengths. These reactions have a rate constant $k_{1}^{\prime }$ (Figure 2, Table 3). Similarly, all reactions under the action of ethylene glycol (EG) proceed at the same rate, with these reactions having a rate constant $k_{2}^{\prime }$ (Figure 2, Table 3). The constants are taken equal to the constants used for calculations in the path of PET synthesis.
  The effective polycondensation rate constant can be determined from Equation (2):
  (2) $$k_{1}^{\prime }=\left[c_{cat}\right]{\cdot k}_{1}=\left[c_{cat}\right]\cdot A\cdot e^{-\frac{E_{a}}{R\cdot T}}$$
  where $k_{1}^{\prime }$ is the effective polycondensation rate constant; k_1_ is the polycondensation rate constant; $\left[c_{cat}\right]$—catalyst concentration, mol/L; A is the pre-exponential factor, 5.66 × 10^8^ L^2^/(mol^2^·min^1^) [42,43]; E_a_—activation energy, 18,500 cal/mol [42,43]; R is the universal gas constant, 1987 cal/mol·K, and T is temperature, K.
  The effective alcoholysis rate constant can be determined from Equation (3):
  (3) $$k_{2}^{\prime }=k_{1}^{\prime }/K$$
  where $k_{2}^{\prime }$ is the effective alcoholysis rate constant, and K is the equilibrium constant, 0.5 [42,43].
4. The volume of the reaction mixture is assumed to be constant, since densities of polyethylene terephthalate, oligoethylene terephthalates, and bis(2-hydroxyethyl) terephthalate are close in magnitude;
5. Mass transfer processes, including the presence of ethylene glycol in the gas phase, are not taken into account. This assumption can be made since the reactors in which PET glycolysis is carried out are usually equipped with reflux condensers, which return ethylene glycol back to the reaction mixture. However, violation of this assumption can lead to an underestimation of the conversion by the model relative to one in the real process, since the reactions involving ethylene glycol are at equilibrium;
6. The concentration of terminal acid groups is taken equal to 0 for all samples. The calculation was made for polyesters and oligoethers with solely hydroxyl end groups;
7. Ester exchange reactions are not taken into account, since their occurrence at the temperatures used makes a significantly smaller contribution than the occurrence of reactions under the action of hydroxyl groups [46];
8. Reactions for the formation of ethers (DEG units), degradation reactions (formation of vinyl groups, aldehydes, and chromophore groups) are not taken into account.

**Figure 3:**
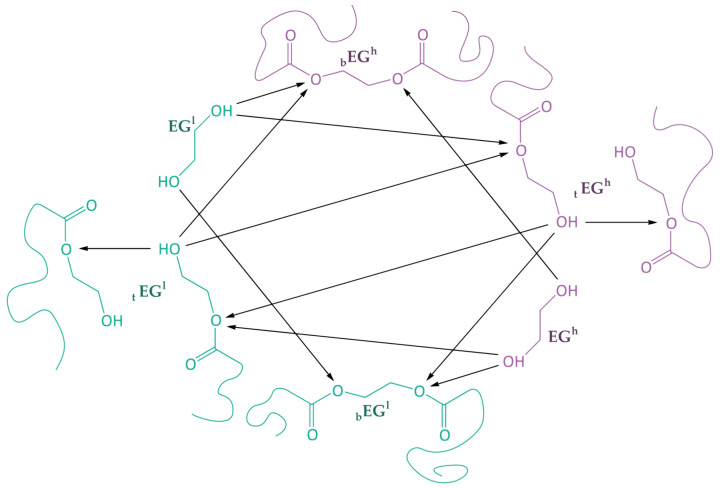
Reactions occurring during PET glycolysis under the action of OET, BHET, and EG, where green—low molecular weight polyester (glycolysis agent), violet— high molecular weight polyester (feedstock).

Thus, the model takes into account the reactions during homogeneous PET glycolysis under the action of oligoethylene terephthalates with terminal hydroxyl groups, bis(2-hydroxyethyl) terephthalate, and/or ethylene glycol, which are shown in Figure 3 and Table 4.

The plug flow reactor (PFR) model was chosen for homogeneous glycolysis of polyethylene terephthalate [42]. The mass balance, which is presented in the form of Equations (4)–(9), takes into account the reactions shown in Table 4, as well as a constant volume, taken equal to 1 L:

(4) $$\frac{d(c_{1})}{dt}={-R}_{1}+R_{4}+R_{5}+R_{6}-R_{7}-R_{10},$$

(5) $$\frac{d\left(c_{2}\right)}{dt}=R_{1}-R_{2}-R_{4}-2R_{5}-R_{6}+R_{7}-R_{9}+{2R}_{10}+R_{11}+R_{12},$$

(6) $$\frac{d(c_{3})}{dt}=R_{2}+R_{5}+R_{9}-R_{10}-R_{11}-R_{12},$$

(7) $$\frac{d(c_{4})}{dt}=R_{1}+R_{2}+R_{3}-R_{4}-R_{8}-R_{11},$$

(8) $$\frac{d(c_{5})}{dt}=R_{1}+R_{2}+R_{3}-R_{4}-R_{8}-R_{11},$$

(9) $$\frac{d(c_{6})}{dt}=R_{3}+R_{6}-R_{7}-R_{8}-R_{9}+R_{12}.$$

The resulting system of differential equations can be solved using numerical methods.

### 3.3. Model Calculations

Verification of kinetic models during their development is carried out by comparing the simulation results with experimental data on the concentration of the components of the reaction mixture or functional groups [44,45]. Since both feedstock and agent polyesters contain the same functional groups, it is impossible to determine their concentrations directly without using expensive deuterated PET, OET, BHET, and/or EG [46]. Therefore, indirect comparison methods can be used to validate the model: molecular weight characteristics of polyesters and the content of bis(2-hydroxyethyl) terephthalate, which is calculated taking into account the Flory–Schulz distribution.

The mass concentration of ethylene terephthalates (excluding EG molecules) in the reaction mixture at the initial moment was determined according to Equation (10):

(10) $$W=M_{PET}\cdot \left({c_{1}+c}_{4}+0.5\cdot {(c_{2}+c}_{5}\right))+M_{EG}\cdot 0.5\cdot {(c_{2}+c}_{5})$$

where W is the mass concentration of ethylene terephthalates (PET, OET, and BHET) in the reaction mixture, g/L; M_PET_ is the molar mass of the PET unit (192 g/mol); and M_EG_ is the molar mass of ethylene glycol (62 g/mol).

The average degrees of polycondensation can be determined from the ratio of terminal and bonded ethylene glycol groups in polyester according to Equations (11)–(14):

(11) $${\bar{X}}_{t}^{h}=\frac{c_{1t}+0.5\cdot c_{2t}}{0.5\cdot c_{2t}}$$

where ${\bar{X}}_{t}^{h}$ is the average degree of polycondensation of feedstock polyester over time t and $c_{1t}$ и $c_{2t}$ are concentrations c_1_ and c_2_ over time t.

(12) $${\bar{X}}_{t}^{l}=\frac{c_{4t}+0.5\cdot c_{5t}}{0.5\cdot c_{5t}}$$

where ${\bar{X}}_{t}^{l}$ is the average degree of polycondensation of glycolysis agent polyester over time t and $c_{4t}$ и $c_{5t}$ are concentrations c_4_ and c_5_ over time t.

(13) $${\bar{X}}_{t}^{o}=\frac{\left(c_{1t}+c_{4t})+0.5\cdot (c_{2t}+c_{5t}\right)}{0.5\cdot (c_{2t}+c_{5t})}$$

where ${\bar{X}}_{t}^{o}$ is the overall average degree of polycondensation of polyester over time t.

The calculations using Equation (13) and the following ones do not take into account the difference in the average degree of polycondensation and molecular weight characteristics between the feedstock polyester and the glycolytic agent. This approach can be preferably used for the glycolysis agent consisting solely of ethylene glycol.

Next, it is necessary to evaluate the molecular weight distribution of polyesters in accordance with the Flory–Schulz equation (Equations (14) and (15)):

(14) $$p_{t}=1-\frac{1}{{\bar{X}}_{t}}$$

where $p_{t}$ is the extent of reaction to be substituted into the Flory–Schulz equation. This variable has no physical meaning for glycolysis.

(15) $$N_{tx}={\left(p_{t}-1\right)}^{2}\cdot {p_{t}}^{x-1}$$

where $N_{tx}$—number fraction of molecules with degree of polycondensation x over time t and x—polycondensation degree, integer value.

To calculate the content of BHET or other fractions of the product, it is necessary to determine the number fraction of ethylene glycol units according to Equations (16) and (17).

(16) $${N(EG)}_{tx}=(x+1)\cdot N_{tx}/\sum_{x=1}^{x=x_{max}}N_{tx}$$

where ${N(EG)}_{tx}$ is the number fraction of ethylene glycol units in the molecule with the degree of polycondensation x over time t (for overall, feedstock, and agent polyesters), $x_{max}$ can be taken equal to 200.

(17) $${N\left(EG\right)}_{tx}^{s}=\frac{{N\left(EG\right)}_{tx}^{h}}{\sum_{x=1}^{x=x_{max}}{N\left(EG\right)}_{tx}^{h}}+\frac{c_{4}+c_{5}}{c_{1}+c_{2}}\cdot \frac{{N\left(EG\right)}_{tx}^{l}}{\sum_{x=1}^{x=x_{max}}{N\left(EG\right)}_{tx}^{l}}$$

where ${N\left(EG\right)}_{tx}^{s}$ is the total summary number fraction of ethylene glycol units in the molecules of feedstock and glycolysis agent with degree of polycondensation x over time t.

**Figure 4:**
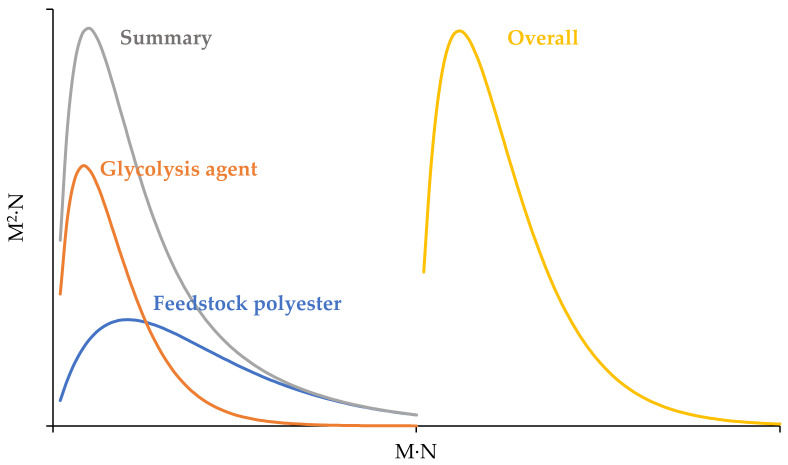
Summary and overall molecular weight distribution.

The calculations using Equation (17) and the following ones take into account the average degree of polycondensation and the molecular weight characteristics of the feedstock polyester and glycolytic agent separately and then summarize them. The molecular weight distribution in this approach is wider than in the calculation of the overall characteristics (PDI^S^ > PDI^O^). A comparison of summary and overall molecular weight distribution is shown in the Figure 4. This approach can be preferably used for a glycolysis agent containing oligoethylene terephthalates.

Equation (18) allows one to obtain data similar to the result of the Flory–Schulz equation for calculating summary characteristics:

(18) $$N_{tx}^{s}=\frac{{N\left(EG\right)}_{tx}^{s}}{x+1}$$

where $N_{tx}^{s}$ is the number fraction of feedstock and glycolysis agent polyesters molecules with degree of polycondensation x over time t.

Similarly, weight fractions can be estimated using the Flory–Schulz equation (Equation (19)) or summing its results for feedstock and agent polyesters (Equation (20)):

(19) $$w_{tx}=x\cdot {\left(p_{t}-1\right)}^{2}\cdot {p_{t}}^{x-1}$$

where $w_{tx}$ is the weight fraction of molecules with degree of polycondensation x over time t (for overall, feedstock, and agent polyesters).

(20) $$w_{tx}^{s}=\frac{w_{tx}^{h}}{\sum_{x=1}^{x=x_{max}}w_{tx}^{h}}+\frac{M_{PET}\cdot \left(c_{4}+0.5\cdot c_{5}\right)+M_{EG}\cdot 0.5\cdot c_{5}}{M_{PET}\cdot \left(c_{1}+0.5\cdot c_{2}\right)+M_{EG}\cdot 0.5\cdot c_{2}}\cdot \frac{w_{tx}^{l}}{\sum_{x=1}^{x=x_{max}}w_{tx}^{l}}$$

where $w_{tx}$ is summary weight fraction of feedstock and glycolysis agent polyester molecules with degree of polycondensation x over time t.

Further, it is possible to evaluate molecular weight characteristics, number average, weight average molecular weight of polyester, and polydispersity index, using Equations (21)–(24):

(21) $$M_{x}=M_{PET}\cdot x+M_{EG}$$

where $M_{x}$ is the molecular weight of the molecule (PET, OET, and BGET) with the degree of polycondensation x.

(22) $$M_{n}=\frac{\sum_{x=1}^{x=x_{max}}{N_{tx}\cdot M}_{x}}{\sum_{x=1}^{x=x_{max}}N_{tx}}$$

where $M_{n}$ is the number average molecular weight.

(23) $$M_{w}=\frac{\sum_{x=1}^{x=x_{max}}{{N_{tx}\cdot M}_{x}}^{2}}{\sum_{x=1}^{x=x_{max}}{N_{tx}\cdot M}_{x}}$$

where $M_{w}$ is the weight average molecular weight.

(24) $$PDI=\frac{M_{w}}{M_{n}}$$

where PDI is the polydispersity index.

The yield was determined through the number fractions of ethylene glycol units according to Equations (25) and (26):

(25) $$W_{tx}=\frac{\left(192\cdot x+62)\cdot (c_{1}+c_{2}+c_{4}+c_{5}\right)}{x+1}\cdot \frac{{N(EG)}_{tx}}{\sum_{x=1}^{x=x_{max}}{N(EG)}_{tx}}$$

where $W_{tx}$ is the mass concentration of molecules with the degree of polycondensation x over time t, g/L.

(26) $$Y_{tx}=\frac{W_{tx}}{W}\cdot 100\%$$

where $Y_{tx}$ is the yield of molecules with the degree of polycondensation x over time t, wt.%.

In addition, the yield can be similarly defined in terms of weight fractions. In this case, the obtained values should differ within the margin of error.

(27) $$Y_{tx}=\frac{w_{tx}^{}}{\sum_{x=1}^{x=x_{max}}w_{tx}^{}}\cdot 100\%$$

(28) $$W_{tx}=Y_{tx}\cdot W/100\%$$

Thus, the main output data of the model, which can be compared with the experimental ones, are the number average and weight average molecular weights; the polydispersity index for summary; overall, feedstock, or agent polyesters; and the yield of bis(2-hydroxyethyl) terephthalate Y_BHET_ calculated for summary and overall polyesters via number or weight fractions.

### 3.4. Homogeneous Glycolysis under the Action of Ethylene Glycol in Solution

A step of 0.1 min was used for the numerical solution of the system of differential equations of the material balance. The temperature conditions were set equal to the experimental ones according to Section 2.3.1; the concentration of the catalyst in terms of dry Sb_2_O_3_ was set equal to 0.048 mol/L, which corresponds to 1 wt.% of the reaction mixture. The initial concentrations used in the calculation are shown in Table 5.

**Figure 5:**
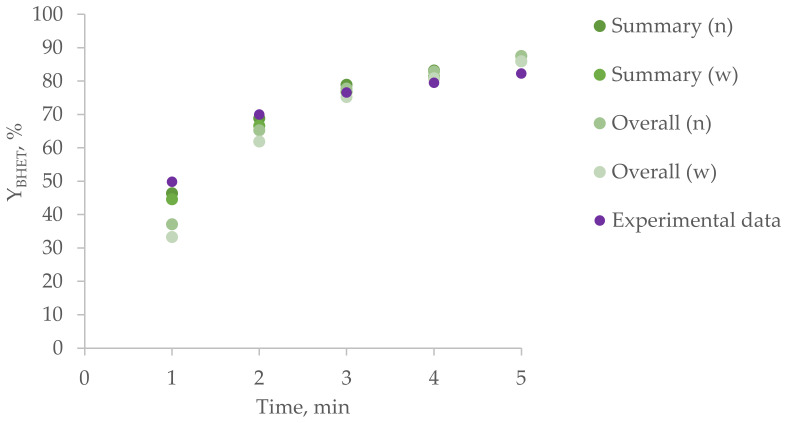
BHET yield for homogeneous glycolysis under the action of ethylene glycol in DMSO.

**Figure 6:**
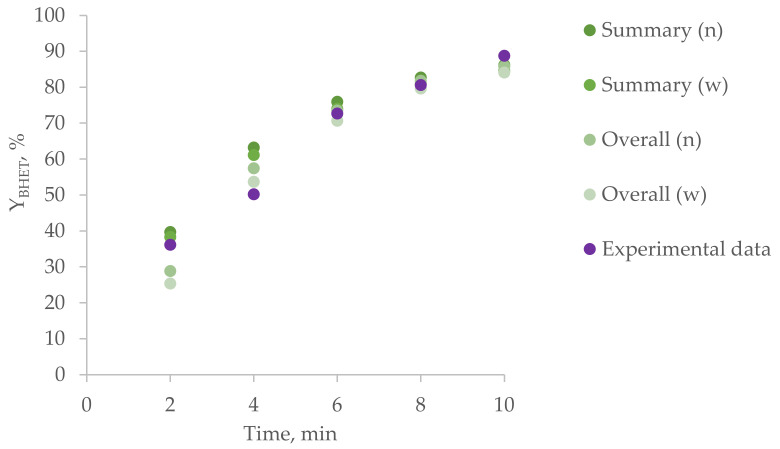
BHET yield for homogeneous glycolysis under the action of ethylene glycol in NMP.

The experimental results are compared with the simulation ones in Figure 5 and Figure 6. Y_BHET_ was calculated for summary and overall polyesters via number (n) or weight (w) fractions.

It can be noted that the simulation results for the summary and overall BHET yield calculations differ significantly at the initial stages, while they practically coincide at subsequent stages. In addition, the model fits the experimental data at the initial stages worse than at subsequent ones, which can be explained by errors in sampling for such a fast reaction or insufficiently rapid achievement of the desired reaction temperature after the introduction of the catalyst solution in ethylene glycol. Another possible reason for the deviation of the calculation results from the experimental data may be a violation of the assumptions made earlier. In particular, the presence of a part of ethylene glycol in the gas phase (assumption 5) with EG being the main agent of glycolysis in this case can lead to a decrease in the conversion and, consequently, a decrease in the BHET yield.

### 3.5. Homogeneous Melt Glycolysis under the Action of BHET

The temperature conditions and the total reaction time were set equal to the experimental ones according to Section 2.3.2. The concentration of antimony trioxide was taken equal to 0.024 mol/L (0.5 wt.% of the mass of the reaction mixture). Initial concentrations are shown in Table 6. Simulation results and experimental data are shown in Table 7.

The simulated number and weight average molecular weights are in agreement with ones obtained experimentally. The calculated polydispersity indices are lower than those obtained experimentally, which may be due to the limitations of using the Flory–Schulz distribution (assumption 1). A potential solution to this discrepancy would be to replace the ideal Flory–Huggins distribution for step-growth polymerization. However, we are not aware of a more suitable molecular weight distribution model. The model also underestimates the concentration of bis(2-hydroxyethyl) terephthalate in the final product. Apparently, this may be due to an overestimation of the reaction rate of two terminal hydroxyl groups with the formation of ethylene glycol. A total of 0.49 and 1.23 g/mol of ethylene glycol are present in the final product according to the simulation at a PET:BHET molar ratio of 1:1 and 1:3, respectively.

### 3.6. Homogeneous Melt Glycolysis under the Action of OET

The temperature conditions and reaction time were set in accordance with the experimental ones (Section 2.3.3) to simulate homogeneous glycolysis under the action of oligoethylene terephthalates with hydroxyl end groups; the catalyst concentration was the same as in Section 3.5. The initial concentrations used in the calculation are shown in Table 8 and the results are shown in Table 9.

The number average and weight average molecular weights and the polydispersity index for PET:OET-1 with a molar ratio equal to 1:1 are significantly higher than calculated ones. This may be due to both the model assumptions and the higher degree of polycondensation of the glycolysis agent OET-1 relative to the theoretical one. BHET yields for glycolysis under the action of oligoesters are small and differ insignificantly, which corresponds to theoretical expectations.

### 3.7. Step-By-Step Homogeneous Melt Glycolysis

Temperature conditions and reaction times for each stage were set equal to the experimental ones (Section 2.3.4). The concentration of antimony trioxide was taken equal to 0.0048 mol/L (0.1 wt.% of the mass of the reaction mixture). The initial concentrations are shown in Table 10. In the simulation, the initial concentrations were not calculated separately for each stage, but the initial concentration for each subsequent stage was calculated based on the final concentrations of the previous stage and the concentration of the agent added. The simulation results and experimental data are shown in Table 11.

All results are in good agreement with experimental data, including previously published data [3] despite the fact that the process is stepwise, and the calculation at each stage was based on the result of the previous stage simulation.

## 4. Conclusions

Thus, the kinetics model of homogeneous poly(ethylene terephthalate) glycolysis has been proposed, which takes into account the reactions of terminal and bound groups of ethylene glycol in polyesters, as well as free ethylene glycol molecules. A method for calculating the number average and weight average molecular weights, the polydispersity index, and the content of bis(2-hydroxyethyl) terephthalate monomer was proposed for the model.

A homogeneous glycolysis in solution under the action of ethylene glycol in DMSO and NMP, homogeneous glycolysis in the melt under the action of bis(2-hydroxyethyl) terephthalate, and homogeneous glycolysis under the action of oligoethylene terephthalate with terminal hydroxyl groups and with a degree of polycondensation equal to 3 were considered. Moreover, step-by-step glycolysis was also considered, where PET interacts with oligoethylene terephthalates, as well as oligoethylene terephthalates with bis(2-hydroxyethyl) terephthalate and ethylene glycol. The model was found to be in good agreement with the experimental data obtained based on the procedures described in the existing literature regarding homogeneous glycolysis.

The deviations in the simulation results do not have a systematic term, with the exception of the polydispersity indices, which were lower than those determined experimentally in all cases. Deviations may be due to the assumptions underlying the model, or errors in the experimental measurements.

In general, the model can be used for further study and optimization of the parameters of homogeneous glycolysis of polyethylene terephthalate under the action of ethylene glycol, bis(2-hydroxyethyl) terephthalate, and hydroxyl-terminated oligoethylene terephthalates. The optimal conditions for homogeneous glycolysis can be determined by comparing the output data of the model (molecular weight characteristics and BHET yield) at various concentrations of the catalyst and reagents, temperature, and reaction time. BHET and OET purified via recrystallization could be polymerized in a similar way to the production of virgin PET or copolyesters. Glycolysis products can be used both separately and in a mixture with BHET based on dimethyl terephthalate or terephthalic acid, with the process being carried out on known equipment for the polyethylene terehpthalate synthesis. A deeper understanding of this process will make it possible to produce recycled polymers with desired properties, as well as to control the properties of polymer-based composites on their basis.

## Figures and Tables

**Table 1:** Step-by-step homogeneous melt glycolysis conditions [3].

Quantity	Reaction Step 1	Reaction Step 2	Reaction Step 3
PET loading, relative mass fraction	100	430	820
OET-1 loading, relative mass fraction	330		
BHET-1 loading, relative mass fraction	0	390	
EG loading, relative mass fraction	0	0	100
Sb_2_O_3_ + EG loading, relative mass fraction	0.86	1.64	1.84
Temperature, °C	250	220	190

**Table 2:** Abbreviations used in the kinetic model, where b—bonded, t—terminal, h—high molecular weight polyester (feedstock), l—low molecular weight polyester (agent).

Name of the Reacting Component	Abbreviation	Source of the Reacting Component	Concentration, g/L
Bonded ethylene glycol	EGhb	Feedstock: Poly(ethylene terephthalate), oligo(ethylene terephthalate), bis(2-hydroxyethyl) terephthalate, and/or ethylene glycol)	c_1_
Terminal ethylene glycol	EGht		c_2_
Ethylene glycol	${EG}^{h}$		c_3_
Bonded ethylene glycol	EGlb	Glycolysis agent: Oligo(ethylene terephthalate), bis(2-hydroxyethyl) terephthalate, and/or ethylene glycol)	c_4_
Terminal ethylene glycol	EGlt		c_5_
Ethylene glycol	${EG}^{l}$		c_6_

**Table 3:** Effective rate constants of reactions according to Figure 2.

Reaction (Figure 2)	Reaction Rate Constant
*n* ≥ 1, m ≥ 0	$k_{1}^{\prime }$
*n* = 0, m ≥ 0	$k_{2}^{\prime }$

**Table 4:** Reactions that occur during PET glycolysis under the action of OET, BHET, and EG, and the corresponding reaction rate equations.

№	Reaction	Reaction Rate Equation
1	EGlt+EGhb→2k1′EGlb+EGht	$R_{1}=2k_{1}^{\prime }\cdot c_{5}\cdot c_{1}$
2	EGlt+EGht→k1′EGlb+EGh	$R_{2}=k_{1}^{\prime }\cdot c_{5}\cdot c_{2}$
3	EGlt+EGlt→k1′EGlb+EGl	$R_{3}=k_{1}^{\prime }\cdot c_{5}\cdot c_{5}$
4	EGht+EGlb→2k1′EGhb+EGlt	$R_{4}={2k}_{1}^{\prime }\cdot c_{2}\cdot c_{4}$
5	EGht+EGht→k1′EGhb+EGh	$R_{5}=k_{1}^{\prime }\cdot c_{2}\cdot c_{2}$
6	EGht+EGlt→k1′EGhb+EGl	$R_{6}=k_{1}^{\prime }\cdot c_{2}\cdot c_{5}$
7	EGl+EGhb→4k2′EGlt+EGht	$R_{7}=4k_{2}^{\prime }\cdot c_{6}\cdot c_{1}$
8	EGl+EGlb→4k2′EGlt+EGlt	$R_{8}=4k_{2}^{\prime }\cdot c_{6}\cdot c_{4}$
9	EGl+EGht→2k2′EGlt+EGh	$R_{9}=2k_{2}^{\prime }\cdot c_{6}\cdot c_{2}$
10	EGh+EGhb→4k2′EGht+EGht	$R_{10}=4k_{2}^{\prime }\cdot c_{3}\cdot c_{1}$
11	EGh+EGlb→4k2′EGht+EGlt	$R_{11}=4k_{2}^{\prime }\cdot c_{3}\cdot c_{4}$
12	EGh+EGlt→2k2′EGht+EGl	$R_{12}=2k_{2}^{\prime }\cdot c_{3}\cdot c_{5}$

**Table 5:** Initial concentrations for the simulation of homogeneous glycolysis under the action of ethylene glycol in solution.

Corresponding Experiment	c_1_, g/L	c_2_, g/L	c_3_, g/L	c_4_, g/L	c_5_, g/L	c_6_, g/L
2.3.1, in DMSO	0.71	0.01	0	0	0	4.38
2.3.1, in NMP	0.43	0.01	0	0	0	3.60

**Table 6:** Initial concentrations for modeling of homogeneous melt glycolysis under the action of BHET.

PET:BHET Molar Ratio	c_1_, g/L	c_2_, g/L	c_3_, g/L	c_4_, g/L	c_5_, g/L	c_6_, g/L
1:1	3.08	0.05	0	0	6.16	0
1:3	1.44	0.02	0	0	8.63	0

**Table 7:** Molecular weight characteristics and BHET yield for homogeneous melt glycolysis under the action of BHET.

PET:BHET Molar Ratio		M_n_	M_w_	PDI	Y_BHET_, %
1:1	Experimental data	530	850	1.60	20.3
	Simulation result	563	839	1.49	14.66 (w) 17.26 (n)
1:3	Experimental data	410	630	1.50	33.8
	Simulation result	446	611	1.37	25.02 (w) 28.50 (n)

**Table 8:** Initial concentrations for homogeneous melt glycolysis under the action of OET modeling.

PET:OET Molar Ratio	c_1_, g/L	c_2_, g/L	c_3_, g/L	c_4_, g/L	c_5_, g/L	c_6_, g/L
1:1	3.41	0.05	0	2.27	2.27	0
1:3	1.66	0.03	0	3.33	3.33	0

**Table 9:** Molecular weight characteristics and BHET yield for homogeneous melt glycolysis under the action of OET.

PET:OET Molar Ratio		M_n_	M_w_	PDI	Y_BHET_, %
1:1	Experimental data	1520	3050	2.00	4.2
	Simulation result	1295	2287	1.77	2.42 (w) 3.05 (n)
1:3	Experimental data	890	1580	1.80	5.7
	Simulation result	933	1567	1.68	4.86 (w) 6.00 (n)

**Table 10:** Initial concentrations for step-by-step homogeneous melt glycolysis modeling.

Reaction Step	c_1_, g/L	c_2_, g/L	c_3_, g/L	c_4_, g/L	c_5_, g/L	c_6_, g/L
1	1.66	0.03	0	3.33	3.33	0
2	0.58	0.33	0.02	2.26	6.46	0.09
3	0.31	0.44	0.08	2.96	4.20	3.07

**Table 11:** Molecular weight characteristics and BHET yield for step-by-step homogeneous melt glycolysis.

Reaction Step		M_n_	M_w_	PDI	Y_BHET_, %
1	Experimental data	890	1590	1.80	5.7
	Simulation result	933	1567	1.68	4.86 (w) 6.00 (n)
2	Experimental data	560	840	1.50	20.0
	Simulation result	524	763	1.45	17.24 (w) 20.11 (n)
3	Experimental data	320	450	1.40	41.6
	Simulation result	393	511	1.30	33.56 (w) 37.40 (n)

## Data Availability

Not applicable.

## References

[1] Kirshanov K., Toms R., Aliev G., Naumova A., Melnikov P., Gervald A. (2022). Recent Developments and Perspectives of Recycled Poly(Ethylene Terephthalate)-Based Membranes: A Review. Membranes.

[2] Benyathiar P., Kumar P., Carpenter G., Brace J., Mishra D.K. (2022). Polyethylene Terephthalate (PET) Bottle-to-Bottle Recycling for the Beverage Industry: A Review. Polymers.

[3] Kirshanov K., Toms R., Melnikov P., Gervald A. (2022). Investigation of Polyester Tire Cord Glycolysis Accompanied by Rubber Crumb Devulcanization. Polymers.

[4] Kirshanov K.A., Toms R.V., Gerval’d A.Y. (2022). Prospects of Polyester Tire Cord Waste Utilization. Kauchuk I Rezina.

[5] Suhaimi N.A.S., Muhamad F., Abd Razak N.A., Zeimaran E. (2022). Recycling of Polyethylene Terephthalate Wastes: A Review of Technologies, Routes, and Applications. Polym. Eng. Sci..

[6] Kirshanov K., Toms R., Melnikov P., Gervald A. (2022). Unsaturated Polyester Resin Nanocomposites Based on Post-Consumer Polyethylene Terephthalate. Polymers.

[7] Singh A.K., Bedi R., Kaith B.S. (2021). Composite Materials Based on Recycled Polyethylene Terephthalate and Their Properties—A Comprehensive Review. Compos. B Eng..

[8] Dasan K.P. (2015). PET Nanocomposites: Preparation and Characterization. Poly(Ethylene Terephthalate) Based Blends, Composites and Nanocomposites.

[9] Celik Y., Shamsuyeva M., Endres H.J. (2022). Thermal and Mechanical Properties of the Recycled and Virgin PET—Part I. Polymers.

[10] Quintero Y.G., Figueroa D.R., Gil H., Zuleta A.A. (2019). Physical and Mechanical Properties of Recycled Pet Composites. Stavební Obz.-Civ. Eng. J..

[11] Rusu M.A.A., Radu S.-A., Moldovan C., Sarosi C., Mazilu (Moldovan) I.A., Rusu L.M. (2019). Mechanical and Structural Properties of Composites Made from Recycled and Virgin Polyethylene Terephthalate (PET) and Metal Chip or Mesh Wire. MATEC Web Conf..

[12] Velásquez E.J., Garrido L., Guarda A., Galotto M.J., López de Dicastillo C. (2019). Increasing the Incorporation of Recycled PET on Polymeric Blends through the Reinforcement with Commercial Nanoclays. Appl. Clay Sci..

[13] Gomzyak V.I., Bychkov N.V., Aduev A.S., Ivanova V.A., Koshelev A.D., Chvalun S.N. (2022). Polymerization of D,L-Lactide in the Presence of Boltorn^TM^ Polyester Polyol. Fine Chem. Technol..

[14] Barnard E., Rubio Arias J.J., Thielemans W. (2021). Chemolytic Depolymerisation of PET: A Review. Green Chem..

[15] Ghasemi M.H., Neekzad N., Ajdari F.B., Kowsari E., Ramakrishna S. (2021). Mechanistic Aspects of Poly(Ethylene Terephthalate) Recycling–toward Enabling High Quality Sustainability Decisions in Waste Management. Environ. Sci. Pollut. Res..

[16] Wu H.-S., Damayanti (2021). Strategic Possibility Routes of Recycled PET. Polymers.

[17] Krisbiantoro P.A., Chiao Y.-W., Liao W., Sun J.-P., Tsutsumi D., Yamamoto H., Kamiya Y., Wu K.C.-W. (2022). Catalytic Glycolysis of Polyethylene Terephthalate (PET) by Solvent-Free Mechanochemically Synthesized MFe2O4 (M = Co, Ni, Cu and Zn) Spinel. Chem. Eng. J..

[18] Du J.-T., Sun Q., Zeng X.-F., Wang D., Wang J.-X., Chen J.-F. (2020). ZnO Nanodispersion as Pseudohomogeneous Catalyst for Alcoholysis of Polyethylene Terephthalate. Chem. Eng. Sci..

[19] Jeong J.-M., Jin S.B., Son S.G., Suh H., Moon J.-M., Choi B.G. (2021). Fast and Facile Synthesis of Two-Dimensional Fe ^III^ Nanosheets Based on Fluid-Shear Exfoliation for Highly Catalytic Glycolysis of Poly(Ethylene Terephthalate). React. Chem. Eng..

[20] Jeong J., Jin S.B., Park H.J., Park S.H., Jeon H., Suh H., Park Y., Seo D., Hwang S.Y., Kim D.H. (2020). Large-Scale Fast Fluid Dynamic Processes for the Syntheses of 2D Nanohybrids of Metal Nanoparticle-Deposited Boron Nitride Nanosheet and Their Glycolysis of Poly(Ethylene Terephthalate). Adv. Mater Interfaces.

[21] Fehér Z., Kiss J., Kisszékelyi P., Molnár J., Huszthy P., Kárpáti L., Kupai J. (2022). Optimisation of PET Glycolysis by Applying Recyclable Heterogeneous Organocatalysts. Green Chem..

[22] Lalhmangaihzuala S., Laldinpuii Z., Lalmuanpuia C., Vanlaldinpuia K. (2020). Glycolysis of Poly(Ethylene Terephthalate) Using Biomass-Waste Derived Recyclable Heterogeneous Catalyst. Polymers.

[23] Liu B., Lu X., Ju Z., Sun P., Xin J., Yao X., Zhou Q., Zhang S. (2018). Ultrafast Homogeneous Glycolysis of Waste Polyethylene Terephthalate via a Dissolution-Degradation Strategy. Ind. Eng. Chem. Res..

[24] Moncada J., Dadmun M.D. (2023). The Structural Evolution of Poly(Ethylene Terephthalate) Oligomers Produced via Glycolysis Depolymerization. J. Mater. Chem. A Mater..

[25] Hu Y., Wang Y., Zhang X., Qian J., Xing X., Wang X. (2020). Synthesis of Poly(Ethylene Terephthalate) Based on Glycolysis of Waste PET Fiber. J. Macromol. Sci. Part A.

[26] Mohammadi S., Enayati M. (2022). Dual Catalytic Activity of Antimony (III) Oxide: The Polymerization Catalyst for Synthesis of Polyethylene Terephthalate Also Catalyze Depolymerization. Polym. Degrad. Stab..

[27] Biros S.M., Bridgewater B.M., Villeges-Estrada A., Tanski J.M., Parkin G. (2002). Antimony Ethylene Glycolate and Catecholate Compounds: Structural Characterization of Polyesterification Catalysts. Inorg. Chem..

[28] Abdullah M.M.S., Al-Lohedan H.A. (2020). Demulsification of Water in Heavy Crude Oil Emulsion Using a New Amphiphilic Ionic Liquid Based on the Glycolysis of Polyethylene Terephthalate Waste. J. Mol. Liq..

[29] Liu Y., Yao X., Yao H., Zhou Q., Xin J., Lu X., Zhang S. (2020). Degradation of Poly(Ethylene Terephthalate) Catalyzed by Metal-Free Choline-Based Ionic Liquids. Green Chem..

[30] Shuangjun C., Weihe S., Haidong C., Hao Z., Zhenwei Z., Chaonan F. (2021). Glycolysis of Poly(Ethylene Terephthalate) Waste Catalyzed by Mixed Lewis Acidic Ionic Liquids. J. Therm. Anal Calorim..

[31] Zhu C., Fan C., Hao Z., Jiang W., Zhang L., Zeng G., Sun P., Zhang Q. (2022). Molecular Mechanism of Waste Polyethylene Terephthalate Recycling by the 1,5,7-Triazabicyclo[4.4.0]Decium Acetate/Zinc Acetate Deep Eutectic Solvent: The Crucial Role of 1,5,7-Triazabicyclo[4.4.0]Decium Cation. Appl. Catal. A Gen..

[32] Marullo S., Rizzo C., Dintcheva N.T., D’Anna F. (2021). Amino Acid-Based Cholinium Ionic Liquids as Sustainable Catalysts for PET Depolymerization. ACS Sustain. Chem. Eng..

[33] Park R., Sridhar V., Park H. (2020). Taguchi Method for Optimization of Reaction Conditions in Microwave Glycolysis of Waste PET. J. Mater. Cycles Waste Manag..

[34] Zahova S., Tsacheva I., Troev K., Mitova V. (2023). Conventional and MW Assisted PET Glycolysis Promoted by Titanium Based Catalyst. Polym. Degrad. Stab..

[35] Bahramian A. (2021). Synergistic Effects of Gamma Irradiation on the PET Surface and Heat Treatment of Hydrotalcite Catalyst Supported by Pt/TiO_2_ Nanoparticles on PET Depolymerization Rate. Surf. Interface Anal..

[36] López-Fonseca R., Duque-Ingunza I., de Rivas B., Flores-Giraldo L., Gutiérrez-Ortiz J.I. (2011). Kinetics of Catalytic Glycolysis of PET Wastes with Sodium Carbonate. Chem. Eng. J..

[37] Viana M.E., Riul A., Carvalho G.M., Rubira A.F., Muniz E.C. (2011). Chemical Recycling of PET by Catalyzed Glycolysis: Kinetics of the Heterogeneous Reaction. Chem. Eng. J..

[38] Sangalang A., Bartolome L., Kim D.H. (2015). Generalized Kinetic Analysis of Heterogeneous PET Glycolysis: Nucleation-Controlled Depolymerization. Polym. Degrad. Stab..

[39] El Mejjatti A., Harit T., Riahi A., Khiari R., Bouabdallah I., Malek F. (2014). Chemical Recycling of Poly(Ethylene Terephthalate). Application to the Synthesis of Multiblock Copolyesters. Express Polym. Lett..

[40] Kirshanov K.A., Gervald A.Y., Toms R.V., Lobanov A.N. (2022). Obtaining Phthalate Substituted Post-Consumer Polyethylene Terephthalate and Its Isothermal Crystallization. Fine Chem. Technol..

[41] Javed S., Fisse J., Vogt D. (2023). Optimization and Kinetic Evaluation for Glycolytic Depolymerization of Post-Consumer PET Waste with Sodium Methoxide. Polymers.

[42] Ha K.-S., Rhee H.-K. (2002). Optimal Reaction Conditions for the Minimization of Energy Consumption and By-Product Formation in a Poly(Ethylene Terephthalate) Process. J. Appl. Polym. Sci..

[43] Kim I.S., Woo B.G., Choi K.Y., Kiang C. (2003). Two-Phase Model for Continuous Final-Stage Melt Polycondensation of Poly(Ethylene Terephthalate). III. Modeling of Multiple Reactors with Multiple Reaction Zones. J. Appl. Polym. Sci..

[44] Kim J.-Y., Kim H.-Y., Yeo Y.-K. (2001). Identification of Kinetics of Direct Esterification Reactions for PET Synthesis Based on a Genetic Algorithm. Korean J. Chem. Eng..

[45] Liu T., Gu X., Wang J., Feng L. (2019). Modeling and Analysis of New Reactor Concepts for Poly(Ethylene Terephthalate) Esterification Process. Chem. Eng. Process.-Process Intensif..

[46] Collins S., Peace S.K., Richards R.W., MacDonald W.A., Mills P., King S.M. (2000). Transesterification in Poly(Ethylene Terephthalate). Molecular Weight and End Group Effects. Macromolecules.

